# Thyroid Autoimmunity: Role of Anti-thyroid Antibodies in Thyroid and Extra-Thyroidal Diseases

**DOI:** 10.3389/fimmu.2017.00521

**Published:** 2017-05-09

**Authors:** Eleonore Fröhlich, Richard Wahl

**Affiliations:** ^1^Internal Medicine (Department of Endocrinology and Diabetology, Angiology, Nephrology and Clinical Chemistry), University of Tuebingen, Tuebingen, Germany; ^2^Center for Medical Research, Medical University Graz, Graz, Austria

**Keywords:** autoimmune thyroiditis, thyroid autoantibodies, thyroid peroxidase, thyroglobulin, thyroid-stimulating hormone receptor, Graves’ orbitopathy

## Abstract

Autoimmune diseases have a high prevalence in the population, and autoimmune thyroid disease (AITD) is one of the most common representatives. Thyroid autoantibodies are not only frequently detected in patients with AITD but also in subjects without manifest thyroid dysfunction. The high prevalence raises questions regarding a potential role in extra-thyroidal diseases. This review summarizes the etiology and mechanism of AITD and addresses prevalence of antibodies against thyroid peroxidase, thyroid-stimulating hormone receptor (TSHR), and anti-thyroglobulin and their action outside the thyroid. The main issues limiting the reliability of the conclusions drawn here include problems with different specificities and sensitivities of the antibody detection assays employed, as well as potential confounding effects of altered thyroid hormone levels, and lack of prospective studies. In addition to the well-known effects of TSHR antibodies on fibroblasts in Graves’ disease (GD), studies speculate on a role of anti-thyroid antibodies in cancer. All antibodies may have a tumor-promoting role in breast cancer carcinogenesis despite anti-thyroid peroxidase antibodies having a positive prognostic effect in patients with overt disease. Cross-reactivity with lactoperoxidase leading to induction of chronic inflammation might promote breast cancer, while anti-thyroid antibodies in manifest breast cancer might be an indication for a more active immune system. A better general health condition in older women with anti-thyroid peroxidase antibodies might support this hypothesis. The different actions of the anti-thyroid antibodies correspond to differences in cellular location of the antigens, titers of the circulating antibodies, duration of antibody exposure, and immunological mechanisms in GD and Hashimoto’s thyroiditis.

## Introduction

Autoimmune diseases (AD) represent a spectrum of disorders caused by inflammation of organs due to production of antibodies against self-structures and cytotoxic action of T cells. Data from Europe, North America, Australia, New Zealand (defined as area 1) and Asia, Middle East, Caribbean, South America (defined as area 2) differ in the reported prevalence (cases/100,000 individuals) of the most frequent AD (1) as follows: Graves’ disease (GD, area 1: 50–626, area 2: 20), Hashimoto’s thyroiditis (HT, chronic autoimmune thyroiditis, autoimmune hypothyroidism, area 1: 300–2,980, area 2: 350), rheumatoid arthritis (RA, area 1: 310–810, area 2: 120–550), type 1 diabetes mellitus (T1DM, area 1: 310–570, area 2: no data), Crohn’s disease (CRD, area 1: 28–201, area 2: 6–113), multiple sclerosis (MS, area 1: 177–358, area 2: 4–101), and Sjögren disease (SD, area 1: 93–3,500, area 2: 330–1,560). The disparity by sex is high in most of these diseases with a female preponderance of ≥85%; only in some childhood-onset AD, such as T1DM, is the risk equal in both sexes. There are three age peaks for AD onset, namely, 8–10 years (juvenile RA, T1DM), 33–50 years (myasthenia gravis, MS, systemic lupus erythematosus, scleroderma, GD), and 52–63 years (HT, SD, adult RA, etc.) (2).

Autoimmune thyroid diseases (AITDs) include several inflammatory thyroid diseases with GD and HT as most frequent forms (3).

## Factors Linked to Thyroid Autoimmunity

Autoimmune thyroid diseases are usually accompanied by the presence of anti-thyroid peroxidase (TPO), anti-thyroglobulin (Tg), and anti-thyroid-stimulating hormone receptor (TSHR) antibodies. Antibodies against thyroid antigens such as carbonic anhydrase 2, megalin, T3 and T4, sodium iodide symporter (NIS), and pendrin have also been detected, although rarely (4).

### Antibodies to Thyroid Antigens

Table 1 provides a comparison of characteristics of anti-TSHR, anti-TPO, and anti-Tg antibodies.

**Table 1:** **Characterization of different anti-thyroid antibodies**.

Parameter	Antibodies		
	Anti-TSHR	Anti-TPO	Anti-Tg
Antigen location	Extracellular	Intracellular	Intrafollicular, low levels in blood circulation
Access of immune cells to antigen	Without tissue destruction	After thyrocyte destruction	With and without tissue destruction
Duration of antigen exposure	Short, low levels (normalization upon treatment)	Prolonged time, intermediate levels (pathologic levels also upon treatment)	Prolonged time, high levels (pathologic levels also upon treatment)
Type of antibody	Oligoclonal, different epitopes	Polyclonal, one domain immunodominant	Polyclonal, different epitopes
Class of antibody	Mainly IgG1, other subclasses to low extent	IgG1, IgG4 > IgG2, IgG3; low levels of IgA	IgG1, IgG4 > IgG2, IgG3; low levels of IgA and IgM (healthy individuals)
Action on neonate	Transplacental passage; transient hyperthyroidism or hypothyroidism with delayed development of thyroid gland	Transplacental passage; potential effects on cognitive development	Transplacental passage; potential effects on cognitive development
Prevalence in AITD	~90% GD; ~10% HT	>80% in GD and HT	>50% in GD and HT
Prevalence in other AD	Usually no expression, one study 18% in T1DM	16–37% RA; 40% T1DM; 12–30% CD	12–23% RA; 30% T1DM; 11–32% CD
Action of antibodies	Stimulating, blocking, apoptosis	Little action *per se*	No defined action
Extra-thyroidal targets	Few, defined effects (GO, GDP), partly known mechanism	Several, ill-defined actions (HE, breast cancer), mechanism of action not known	No specific targets identified
Action in breast cancer progression	No protective effect	Potential protective effects	Potential protective effects

*TSHR, thyroid-stimulating hormone receptor; TPO, thyroid peroxidase; Tg, thyroglobulin; CD, celiac disease; GD, Graves’ disease; GDP, Graves’ dermopathy; GO, Graves’ orbitopathy; HT, Hashimoto’s thyroiditis; RA, rheumatoid arthritis; T1DM, type 1 diabetes mellitus; AITD, autoimmune thyroid disease; AD, autoimmune diseases; HE, Hashimoto’s encephalopathy*.

#### Thyroid-Stimulating Hormone Receptor

After expression on the thyrocyte cell surface, the TSHR undergoes cleavage within the “hinge” region at two or more sites; loss of a C-peptide like region leads to an extracellular A-subunit linked by disulfide bonds to the B-subunit (comprising the remainder of the hinge region, transmembrane, and cytoplasmic tail). Subsequently, some A-subunits are shed. Substantial evidence suggests that the shed A-subunit (rather than the holoreceptor) is the autoantigen initiating and/or driving the autoimmune response to the TSHR in GD [for a recent review, see Ref. (5)]. As shown by crystallization, stimulating TSHR monoclonal antibody M22 and TSH blocking monoclonal antibody K1-70 (both of human origin) bind to closely overlapping epitopes at the amino terminus (6). In addition, neutral antibodies directed against the “hinge” region exist (7). These antibodies can induce generation of oxidative radicals and induce apoptosis. The balance between stimulating TSHR and neutral antibodies can provide a balance between thyrocyte proliferation and apoptosis. DNA released from apoptotic cells stimulates the immune response. Since shedding of A-subunits occurs in all humans, the presence of exogenous and endogenous factors (see Environmental (Exogenous) Factors and Endogenous Factors) is mandatory for the development of GD (5).

The biological action of autoantibodies against TSHR was the reason for their discovery by Adams and Purves (8). These authors were the first to identify a molecule with similar action to TSH and termed it long-acting thyroid stimulator (LATS) due to its prolonged effect. LATS was later identified as an immunoglobulin G and later found to be an antibody against TSHR.

Anti-TSHR antibodies are found in 90% of GD patients, 0–20% HT, and 10–75% of atrophic thyroiditis patients (9). Other studies noted TSHR antibodies in around 10% of HT patients (10, 11). Stimulating antibodies can be oligoclonal and belong to IgG1 class, while blocking antibodies are polyclonal and not restricted to a specific subclass (12, 13). The pleiotropic action of anti-thyroid antibodies is typical for anti-TSHR antibodies. Stimulatory antibodies are detected in 73–100% and blocking anti-TSHR antibodies in 25–75% of GD patients (14–16). The variations in the detection appear to be linked to the type of assay that has been used. TRAb usually refers to any type of antibody interacting specifically with the TSHR. When assessed by competitive binding assay, the TSHR antibodies are called TSHR-binding inhibitory immunoglobulins. By contrast, cell-based bioassays measure either TSHR stimulatory antibodies or TSHR-stimulating immunoglobulins, or alternately TSHR-blocking antibodies (TBAb) or TSHR-blocking immunoglobulins (17). Although all studies support the higher prevalence of stimulatory antibodies [for instance, Ref. (18)], the presence of only blocking TSHR antibodies may cause myxedema (19). High TSHR antibodies at diagnosis and/or positive TSHR antibodies at cessation of therapy suggest a high likelihood of relapse, mostly within the first 2 years. The levels can help to identify patients that need definitive therapy (radioiodine or surgery) (20). Anti-TSHR antibodies can cross the placental barrier and may induce transient hyperthyroidism in the neonate (21). Since half-life of IgG is ~3 weeks, symptoms disappear gradually. Blocking antibodies may also cause transient hypothyroidism with delayed development of the neonate thyroid gland.

#### Thyroid Peroxidase

Thyroid peroxidase is a poorly glycosylated membrane-bound enzyme, responsible for iodine (I_2_) oxidation and iodination of tyrosyl residues of the Tg molecule (22). It had been termed microsomal antigen based on its intracellular localization. Antibodies react against conformational epitopes at the surface of the molecules and against linear epitopes (23). Polyclonal antibodies from healthy individuals and patients are directed against the same epitopes. Anti-TPO antibodies from healthy subjects did not block TPO activity or interfere with the blocking activity of anti-TPO antibodies from AITD patients (24), while anti-TPO antibodies from AITD patients can fix complement, destroy thyrocytes, and act as competitive inhibitors of enzymatic activity (25). These antibodies can be of any class of IgG, although some studies indicated a higher prevalence of IgG1 (70%) and IgG4 (66.1%) compared to IgG2 (35.1%) and IgG3 (19.6%) (26). Low levels of IgA antibodies have also been reported (21). Anti-TPO antibodies are more common than anti-Tg antibodies and more indicative for thyroid disease (21). Anti-TPO antibodies are inductors of oxidative stress evidenced by decreased antioxidant potential, advanced glycosylation products and oxygen metabolites in blood (27). However, their contribution to thyroid damage compared to T cell and cytokine-mediated apoptosis is minor (28). Anti-TPO antibodies are detected in 90–95% of AITD patients, 80% of GD, and 10–15% of non-AITD patients (9). While anti-TPO antibodies may act cytotoxic on thyrocytes in HT they do not have an established role in GD (29). Anti-TPO antibodies are able to cross the placenta barrier to variable extent, but the effect on the neonate is unclear. Concerns on a potential negative effect on cognitive development of the offspring have not been confirmed so far (21).

#### Thyroglobulin

Thyroglobulin is a large (600 kDa) glycoprotein consisting of dimers and containing on average 2–3 molecules of T4 and 0.3 molecules T3. The molecule is heterogeneous regarding hormone content, glycosylation, and size. The production of antibodies against Tg can be induced by massive destruction of the thyroid gland, but high Tg levels in blood do not *per se* induce antibody production (4). Out of the 40 epitopes that have been identified, 6 according to some authors and 1–2 according to others are immunogenic (30, 31). Antibodies against Tg differ between healthy subjects and AITD patients in that polyclonal antibodies are seen in normal subjects and oligoclonal antibodies in AITD patients (4). Antibodies in healthy subjects and AITD patients differentially recognize mainly two conformational epitopes of the molecule (32). Pattern of anti-Tg antibodies are similar in GD and HT patients and similar in healthy individuals and patients with TC (33). In general, low levels of self-antigens induce tolerance (34). It has been hypothesized that normal blood levels of Tg induce self-tolerance in T cells but not in B cells. B cells that recognize Tg arrest their migration in the T cell zone of peripheral lymphoid tissues but do not interact with CD4 helper cells. The lack of interaction prevents the B cells from migrating out of the T cell zones into the follicles, and they undergo apoptosis. As a consequence of the B cell activity, healthy individuals have very low, usually below detection threshold levels of anti-Tg antibodies. In the presence of higher Tg levels after tissue damage, changed conformation of the Tg molecule due to high I_2_ levels, and supernormal TSH levels, the anti-Tg antibody titers become abnormal (31). Administration of I_2_ induced antibody production in 8–20% of subjects, together with intra-thyroidal lymphocyte infiltration in some of the patients (35). The proposed mechanisms are either antibody formation due to massive release of antigens following thyrocyte destruction or generation of new epitopes by a changed and more immunogenic conformation of the Tg molecule with high I_2_ content. The effects of I_2_ on immune responses of Tg and TPO antigens in thyroid autoimmunity might not be completely the same. On the basis that salt intake is the main source of I_2_, universal salt iodization has been introduced as protective measure against goiter. Excessive I_2_ intake, defined as table salt I_2_ concentrations of 40–100 mg/kg for 5 years, increased thyroid autoimmunity (36).

Anti-Tg antibodies do not fix complement because the epitopes are too widely spaced to allow cross-linking. Furthermore, anti-Tg antibodies in GD belong mainly to the IgG4 class, which is not complement binding (37). Low levels of IgA antibodies have also been reported (21). IgM antibodies against Tg have been reported to 1% in healthy individuals. The functional consequence of anti-Tg antibodies is not clear as they do not cause thyroid cell destruction. Circulating antibodies could be detected in about 10% of healthy young subjects and 15% of people >60 years of age. Among HT patients, antibody prevalence was 60–80% and in 50–60% in GD patients. Another study identified anti-Tg antibodies in 70–80% of AITD patients, 30–40% of GD patients, and 10–15% of patients with non-thyroid immune disorders (9). Anti-Tg antibodies can cross the placenta barrier, but the effect on the neonate is unclear (21).

The distribution among the classes of antibodies against Tg has been reported differently. IgG1 and IgG4 were the most important classes in GD and HT patients according to one study, while other authors reported distribution between IgG1, IgG2, and IgG4 classes. Interestingly, distribution differed between GD and HT patients; IgG4 class was dominant in patients with GD and IgG2 class in HT patients (37). This different distribution may reflect the different type of immune action taking place in the thyroid.

#### Other Thyroid Antigens

Antibodies against other thyroid autoantigens are not determined routinely since their incidence is much lower and their physiological role unclear. Data obtained by immunization of mice and binding of patient sera to stably transfected COS 7 cells expressing high levels of NIS indicate that the antibodies did not display marked inhibiting effect on iodide uptake (38). Macro-TSH is the result of the binding of anti-TSH antibodies to TSH and results in a high-molecular protein complex with low TSH bioreactivity. Incidence increases with age, and altered TSH antigenicity or decrease in autoimmune tolerance have been proposed as pathogenic mechanism (39).

In summary, prevalence of anti-TPO and anti-Tg antibodies is high in patients with GD and HT, while anti-TSHR antibodies are common in GD patients but relatively rare in patients with HT. This may suggest that anti-TSHR antibodies are produced under more specific situations than the other antibodies. This difference is also reflected in some factors that have opposite effects in GD and HT, like, for instance, smoking and stress (see Factors with Opposite Effects on GD and HT). There are additional differences in development and manifestation of the diseases. GD is usually characterized by rapid onset of the symptoms and is, except for elderly people with less typical symptoms, diagnosed and treated quite fast (40). Established treatments normalize titers of TSHR antibodies in adults within 2 years, while treatment of children and adolescents requires longer treatment times (41, 42). HT develops gradually over months and years with very high antibody titers in some patients (43). Symptoms can be mild, and patients might not seek medical advice. Even when treatment has been initiated, titers of anti-TPO antibodies decrease only slowly (e.g., over 5 years) upon treatment with levothyroxine, and anti-TPO antibody titers remain in the pathological range (44). Normal anti-thyroid antibody titers are lower for anti-TSHR antibodies than for anti-TPO and anti-Tg antibodies. Exact values cannot be compared directly since sensitivities of the assays differ, but the range of >1.75 U/ml for anti-TSHR, >35 U/l for anti-TPO, and >20 U/l for anti-Tg according to most laboratories (45) can serve as approximate indication that titers are markedly different.

### Environmental (Exogenous) Factors

**Figure 1:**
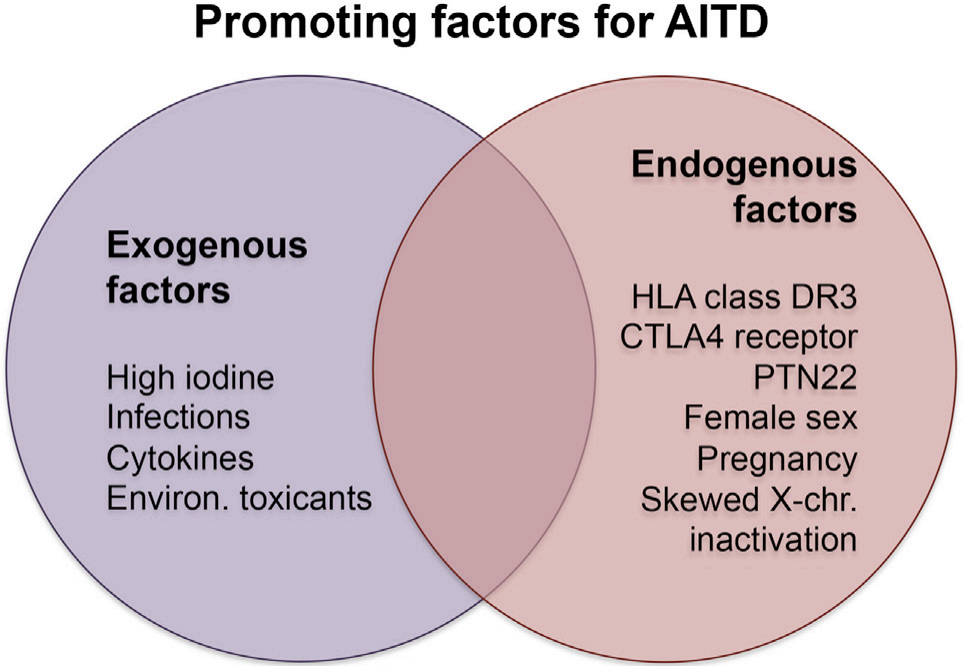
**Promoting factors in Hashimoto’s and Graves’ disease**. Abbreviations: chr., chromosome; CTLA4, cytotoxic T-lymphocyte-associated protein 4; Environ., environmental; PTN22, protein tyrosine phosphatase, non-receptor type 22.

Exogenous and endogenous factors contribute to the risk of developing AITDs. Major exogenous causative factors are infections, intake of particular substances, and radiation, while endogenous factors are mainly sex and genetic disposition (Figure 1). Other factors have opposite effects in the development of GD and HT or protect rather than promote the manifestation of AITD.

#### Factors with Opposite Effects on GD and HT

Exogenous factors include *smoking*, which has a protective effect on HT incidence by lowering anti-Tg and anti-TPO antibody levels, while favoring the development of GD (odd’s ratio, OR 3.3). Opposing effects in AD affecting the same organs have also been reported for inflammatory bowel diseases. Smoking protects against ulcerative colitis but induces worsening of CRD. Recent studies suggest that smoking acts through modulation of the phenotype of dendritic cells, which are involved in the activation and differentiation of T cells (46). It might be speculated that a similar mechanism also causes the different effect of smoking on HT and GD. *Stress* or major life events can increase the prevalence of GD, but not of HT (47).

#### Protective Factors

*Alcohol intake*, which protects against several AD, also decreases the incidence of GD and of HT; abstainers have a 2.17 higher risk than non-abstainers (48, 49). High doses of alcohol consumption can directly suppress immune responses, and alcohol abuse is associated with an increased incidence of a number of infectious diseases (50). Moderate alcohol consumption seems to have a beneficial impact on the immune system compared to alcohol abuse or abstinence, but the mechanisms behind the protective effect are not clear. *Selenium* supplementation does not consistently decrease anti-TPO antibody levels, which might be due to different baseline concentrations of both selenium and anti-TPO antibodies (51). *Vitamin D* supplementation has the reputation of improving immunological function, but studies on vitamin D in AITDs are not conclusive (52); insufficient matching of all patients’ parameters was suspected to potentially hide an existing correlation.

#### Promoting (Worsening) Factors

High oral *I*_2_ intake (~400–600 μg/day) increases the immunogenicity of Tg by creating new epitopes or unmasking cryptic epitopes. These levels, on the other hand, are lower than the short-term treatment for thyrotoxicosis with 250 mg every 6–8 h (53). Iodine usually refers to any form of the molecule including molecular I_2_, I_2_ salts, sodium iodide and potassium iodide (NaI and KI), sodium iodate (NaIO_3_), and lipids or proteins containing I_2_ moieties (iodotyrosin, iodolactone) (54). The human body on the average contains 25–50 mg I_2_, 50–70% of which is stored in extra-thyroidal tissues, especially in the gastrosalivary pool. NaIO_3_ is dissolved in the stomach and releases I_2_, which can be absorbed in the small intestine. In the blood, I_2_ is transported as iodide and can enter cells that express NIS. Only certain cells, for example, breast cancer cells, can take up I_2_ by facilitated diffusion. I_2_ and iodide differ in their antioxidant properties; I_2_ has a 10-fold greater antioxidant potential than ascorbic acid and 50-fold that of iodide (55). Common supplements contain either iodide alone or a mixture of I_2_ and iodide. While the salt carries a great risk for interference with thyroid function, I_2_ is more effective in breast pathologies, such as fibrocystic syndrome and mastalgia. Lugol’s solution (5% I_2_/10% kalium iodide) has a net anti-estrogenic effect. Natural food, fish, and seaweed contain mainly I_2_, but the contents may vary between 1.6 µg/g and 2.9 mg/g. Despite their different physiological effects, all forms of I_2_ have the potential to induce or exacerbate autoantibody formation. In addition, increased I_2_ concentrations can also induce oxidative stress by increased activation of TPO (56). Membrane damage by oxidative stress can cause release of intracellular thyroid antigen and lead to the expression of intercellular adhesion molecule 1 (ICAM-1) by thyrocytes. Furthermore, excess iodide may promote apoptosis by tumor necrosis factor-related apoptosis-inducing ligand and death receptor (DR) 5 expression. Other effects of I_2_ include augmentation of dendritic cell maturation, increase of T cells and antibody production by B cells, and increasing cytokine secretion (28). Iodinization increased the prevalence of anti-TPO antibodies in the population from 14.3 to 23.8% and for anti-Tg antibodies from 13.7 to 19.9% (57). It also increased apoptosis induction *via* the Fas-mediated pathway and downregulation of Bcl-2, CD8^+^ T cell-mediated cytotoxicity by enhanced ICAM-1 expression increasing cell–cell binding, and gap junction changes by downregulation of connexin 43. In the latter case, gap junctions become less tight due to the downregulation of the protein, and TPO antibodies can access TPO at the apical plasma membrane of the thyrocytes and activate complement.

*Infection* with viruses, such as hepatitis C (HCV), hepatitis E, parvovirus, rubella, herpes simplex, Epstein–Barr, or human T lymphotropic virus type 1 (HLTV1), could be involved in the pathogenesis of AITDs (58). The release of IL-8 by thyroid cells infected with HCV as pathogenic mechanism has been demonstrated in cell culture (59). Gut microbiota can trigger HT, and enteroviruses have been identified in thyroid tissues. *Yersinia enterocolitica* antibody cross-reactions with TSHR are assumed to induce GD symptoms (57). This effect has been explained by molecular mimicry, which is defined as the phenomenon whereby exposure to a particular peptide epitope in the environment stimulates antibody production against an endogenous antigen containing the identical amino acid sequences (60). The role of commensal bacteria to the development of HT is not yet proven. Enteropathy (leaky gut syndrome) with increased intestinal permeability and intraepithelial lymphocyte infiltration may also increase the risk for developing thyroid autoimmunity, but further studies are needed (61). Infection with HLTV1 and with *Helicobacter pylori* have been linked to AITD by either thyrocyte damage or cytokine secretion (62). Animal experiments proved the mediator role of interferon-alpha in the induction of inflammatory thyroid destruction (63). Pathways associated with apoptosis and autophagy were not induced. Although contact with specific pathogenic and commensal viruses and bacteria can trigger the manifestation of GD and HT, lack of infections (termed “hygiene hypothesis”) also increases the risk for allergies and AD, such as AITD (64).

Specific *cytokines* such as interferon-gamma (IFN-γ), IL-2, and granulocyte–macrophage colony-stimulating factor promote the development of AITD (65). In a similar way, treatment with the anti-programmed death 1 receptor monoclonal antibody pembrolizumab interfered with thyroid function causing elevation of anti-thyroid antibodies accompanied by AITDs resulting in either hypo- or hyperthyroidism (66).

Epidemiological studies showed that individuals exposed to *environmental toxicants*, such as polyaromatic hydrocarbons, including polychlorinated biphenyls and polyhalogenated biphenyls, had elevated circulating antibody levels against TPO and Tg (67). Furthermore, drugs, like, for instance, lithium in the treatment of bipolar mood disorders, precipitate AITD (68). *Radiation* (therapeutically or environmental) may induce thyrocyte damage and stimulate the development of AITD by release of thyroid antigens (69).

A broad panel of exogenous factors may contribute to the manifestation of AITDs. Some of these thyroiditis forms are also regarded as separate entities, such as radiation-induced (radio-active iodine, beam radiation) thyroiditis and drug-induced (amiodarone, lithium, interferon-α, cytokines) thyroiditis.

### Endogenous Factors

*Genetic effects* can explain 79% of the manifestations of GD, and anti-thyroid antibody formation is positively correlated at a level of 73% in twins (70). Human leukocyte antigen (HLA) class II antigens are thought to contribute 20% of these genetic risk factors. Association with GD was around 30% for cytotoxic T-lymphocyte-associated protein 4 (CTLA4). CD40 can increase HLA and CTLA4 risk. Protein tyrosine phosphatase, non-receptor type 22 (PTN22), was associated with AITDs with an OR of 1.41–3.65 depending on geographic region and race (71). Some *HLA alleles* have a higher affinity for autoantigenic thyroid peptides than others. Polymorphisms in HLA class II DR3 predispose to GD, while for HT various HLA alleles have been reported such as DR3, DR5, DQ7, DQB1, DQw7, and others. Together with the binding of the T cell receptor to the HLA–antigen complex, activation of the T cell immune response requires binding of co-stimulatory ligands such as B7 on antigen-presenting cells (APC) to the CD28 receptor on the T cell. However, B7 also binds the *CTLA4* (*CD28*), which inhibits T cell proliferation to control rampant immune responses; several CTLA4 polymorphisms have been identified in HT and GD patients, mostly single nucleotide polymorphism (SNP). SNPs are also associated with antibody production (28). Certain of these CTLA4 polymorphisms result in a greater proliferative response of the T cells, which might explain associations with AD. SNPs in the *PTN22 gene* enable efficient inhibition of T cell activation and impaired thymic deletion of autoreactive T cells in combination with inhibition of regulatory T cells. This was suggested as reason why this might lead to AD. Additional SNPs have been identified in Tg genes, the vitamin D receptor, IL-4, transforming growth factor-beta (TGF-β), FoxP3, and the tumor necrosis factor-alpha (TNF-α) gene. Genomic imprinting (the activation of maternal or paternal genes) has not been well studied in AD, but the Tg promoter appears to be a candidate for epigenetic effects (silencing or activation of genes) in AITDs (72).

*Female preponderance* is seen in all AD. AITD is one example where this is very pronounced, and many studies aimed to understand the underlying mechanisms focusing first on differences in the immune system. Females have similar numbers of lymphocytes but higher antibody production by B cells. In addition to that, females have stronger humoral and cellular immune responses, higher CD4^+^ T cell levels after immunization, and lower susceptibility to various bacterial infections (73). Typically, only herpes simplex type 2 infections are more common in females than in males. One reason for the observed differences might be the prominent immune modulatory effects of estrogens. Immune cells carry receptors for estrogen, testosterone, and progesterone (74). Estrogen decreases the CD4^+^/CD8^+^ T cell ratio and TNF-α cytotoxicity in T cells and increases immunoglobulin secretion, B cell survival, and polyclonal activation of B cells as well as IgG and IgM production in peripheral blood mononuclear cells. High estrogen levels decrease Th1 pro-inflammatory pathways and increase Th2 anti-inflammatory pathways. Progesterone in general acts in an anti-inflammatory manner by inhibition of macrophage activation, nitric oxide production, and IFN-γ production by NK cells (75).

The higher prevalence of AD in patients with structural X-chromosome defects and monosomy implied a crucial role of the X-chromosome in autoimmune reactions. In women, one of the two X-chromosomes is inactivated in every cell. Usually, the ratio of inactivation is 50:50 for paternal and maternal X-chromosomes. However, in some women a skewed inactivation is seen that could lead to insufficiently high levels of specific antigens to induce self-tolerance. Consistent with this idea *skewed X-chromosome inactivation* has been preferentially detected in women with AITD with a correlation of OR 2.54 for GD and 2.40 for HT (57). The correlation of skewed X-chromosome inactivation and TPO antibody level was stronger in dizygotic than in monozygotic twin supporting the assumption that the inactivation is more important for the manifestation of the disease than the genes themselves. The Y-chromosome is less important for survival because it harbors only a few genes. Nevertheless, loss of the Y-chromosome has been identified in GD and HT patients and is accompanied by reduced testosterone levels. The few genes not related to male fertility are X-chromosome homologs with relevant roles in immune function (76). The loss could cause haplo-insufficiency similar to X-chromosome loss, and an imbalance for the alleles shared with the X-chromosome could emerge. An additional reason might be the relationship between thyroid and testosterone *via* regulation of sex-hormone binding globulin (77). High thyroid hormone levels increase the levels of free testosterone with subsequent physiological effects. Alterations in hormone levels such as increases in testosterone in females with polycystic ovary syndrome increase the prevalence of AITDs (78).

*Pregnancy* has a marked impact on AD, whereby the number of regulatory T cells is increased in gravidity and levels of anti-thyroid antibodies decreased. This decrease is usually transient, and a rebound effect of the antibody levels is seen 6 weeks postpartum (28). The postpartum period is a risk for onset of GD, and postpartum thyroiditis may lead to HT. Postpartum thyroiditis is defined as destructive thyroiditis within 1 year of parturition. The disease is characterized by transient hypothyroidism or hyperthyroidism or hyperthyroidism followed by hypothyroidism. Patients often recover, but there is up to a 50% risk of developing permanent thyroid disease with time (79). Previous pregnancy as a risk factor for AITD could be due to fetal microchimerism, i.e., the phenomenon that fetal cells and antigens are transferred to the mother. Fetal microchimeric cells have been found particularly in AITD patients. The fetal cells were cytotoxic T cells able to mitigate graft-versus-host reactions (75). The more fetal cells show similarities with the maternal cells, the more likely they have the potential to mediate such reactions. Although pregnancies ≥1 have an OR of 1.1–1.8 for AITD compared to nulliparous women there is still no proof for the hypothesis that fetal microchimerism causes HT (80). Furthermore, parity is not linked to increased antibody levels in all studies.

### Immunologic Processes in AITD

**Figure 2:**
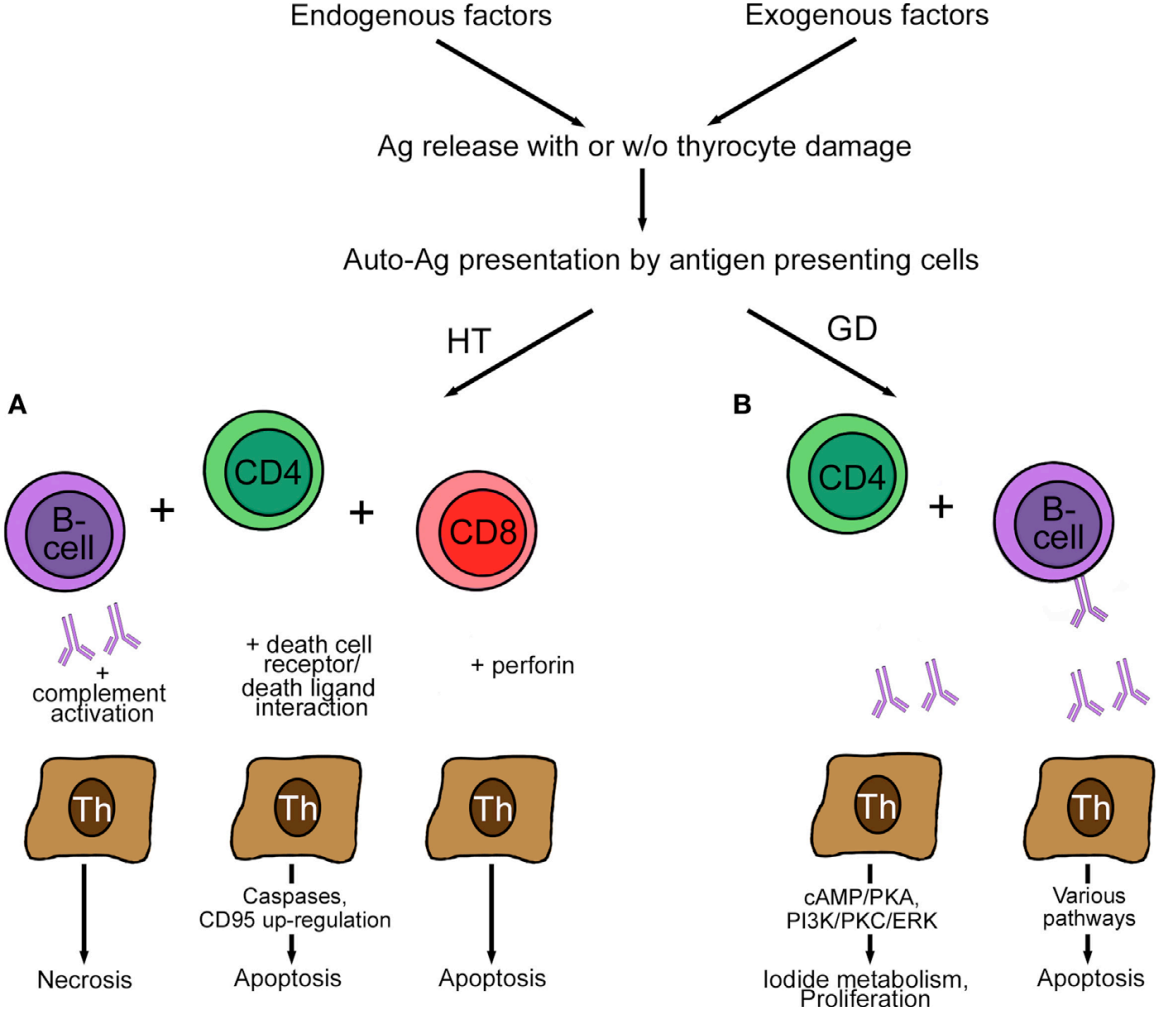
**Pathogenesis of Hashimoto’s disease (A) and GD (B)**. **(A)** Autoreactive CD4^+^ T cells in HT induce antibody production by B cells. The antibodies bind to the basal membrane of the thyroid follicle, activate complement, and induce necrosis of thyrocytes. The activation of cytotoxic CD8^+^ T cells leads to the induction of apoptosis by action of perforin. Finally, the expression of Fas (CD95) and FasL (CD95L) by thyrocytes perpetuates HT. **(B)** Autoreactive CD4^+^ T cells in GD induce only anti-thyroid-stimulating hormone receptor antibody-producing B cells. These antibodies act stimulatory by increasing I_2_ metabolism (cAMP/PKA) and promoting proliferation and survival (PI3K/PKC/ERK) of thyrocytes. Blocking antibodies are characterized by lack of effect (not shown), and neutral antibodies activate various pathways, such as PI3K/Akt, mTOR/p70S6K, and MAPK/ERK1/2 and induce thyrocyte apoptosis. Abbreviations: GD, Graves’ disease; HT, Hashimoto’s thyroiditis; ERK, extracellular signal-regulated kinase; MAPK, mitogen-activated protein kinase; p70S6K, ribosomal protein S6 kinase beta-1; PKC, protein kinase C; PI3K, phosphatidylinositol 3,4,5, triphosphate kinase; PKA, protein kinase; mTOR, mammalian target of rapamycin.

The basic differences between the two most common AITDs, HT and GD, is the reaction to the deregulated immune system. Several excellent reviews are available on the mechanisms of AITD [examples are Ref. (57, 72, 81), which will be only presented in cursory way in this review in Figure 2]. Presentation of autoantigens *via* MHC complex/self-peptide complexes by APC and recognized in the periphery by thymus-selected autoreactive CD4^+^ T cells is the initial step in the pathogenesis of AITDs (82). Reduction of immune tolerance by various mechanisms and changes in the thyroid microenvironment are contributing factors in the development of GD and HT. Despite intense research, the exact mechanism of HT is not completely clear, but the following mechanisms are proposed (83). Decreased function of regulatory T cells, increased activity of follicular T helper cells, release of DNA fragments, and alteration in miRNA profile initiate and perpetuate HT. The number of regulatory T cells plays an important role, and their number increases upon levothyroxine treatment. The hypothesized mechanism of regulatory T cells is the suppression of lymphocyte differentiation to Th1 cells (84). Th17 cells are a newly identified CD4^+^ subgroup of T helper cells, which were found to be increased in the blood of children with untreated HT but not in GD patients. Double-stranded DNA released from the nuclei of damaged thyrocytes is actively taken up or leak into neighboring cells. Extra-chromosomal histone (H2B) recognizes the misplaced DNA and induces the release of pro-inflammatory cytokines *via* translocation of interferon regulatory transcription factor and nuclear factor “kappa-light-chain-enhancer” of activated B cells (NFκB) into the nucleus. As a result of the release of the cytokine/chemokine MHC expression by thyrocytes and lymphocytes infiltration takes place. Activated T and B cells and DR-mediated apoptosis initiate and perpetuate the autoimmune reaction in HT (85). Self-reactive CD4^+^ T cells recruit CD8^+^ cytotoxic T cells, which cause thyrocyte death by release of granzyme and perforin (Figure 2). Antibodies secreted by B cells are deposited at the basal membrane of the thyroid follicle, activate complement system, and thereby induce necrosis. Increased levels of the cytokines IFN-γ and IL-12 (Th1 cytokines) induce TNF-α, IL-1β, IL-2, and CD40L, which cause apoptosis of thyrocytes but not of infiltrating lymphocytes. Apoptotic death of thyrocytes can occur in a paracrine way by expression of cell DR (Fas, apoptosis protein 1, CD95) and death ligands (FasL, CD95L).

In contrast to HT, self-reactive CD4^+^ lymphocytes in GD recruit only TSH-reactive B cells. Secretion of IL-4 (a Th2 cytokine) together with IL-5, IL-6, IL-10, IL-13, and CD40L causes apoptosis of the infiltrating lymphocytes and proliferation of thyrocytes resulting in GD. Lymphocytes resident in the thyroid, not lymphocytes in lymphoid organs, produce the anti-thyroid antibodies. Usually, when the thyroid is removed, the production of autoantibodies ceases.

Antibodies reach the blood stream *via* transepithelial transport by uptake combined with megalin (receptor) and release into the extracellular space or by disruption of the thyroid follicles. Alternatively, secretion by transformed thyrocytes may occur (4). The nature of the immune reaction differs in different AITDs with mainly T cell-mediated autoimmunity in HT and primary humoral response in GD (86). In reality, there are mixed Th1/Th2 responses in both GD and HT and, although the diseases have different phenotypes, GD and HT may coexist in the same thyroid gland, and some individuals progress from one to the other. The transition from GD to hypothyroidism due to HT can occur, while the development of GD from HT is rare (87).

## Anti-Thyroid Antibody Types: The Key to the Diagnosis

Increased levels of anti-thyroid antibodies usually accompany AITDs, and their detection may support its diagnosis. Manifestations of the main AITDs are described in the following sections.

### Hashimoto’s Thyroiditis

Hashimoto’s thyroiditis may lead to manifest hypothyroidism, but most patients have subclinical hypothyroidism with increased TSH and normal thyroid hormone levels. There are different variants of HT, termed fibrous, fibrous atrophic (Ord’s disease), or goitrous forms, and IgG4 thyroiditis (88). Based on debates that Riedel thyroiditis is not primarily a thyroid disease but rather a manifestation of the systemic disorder multifocal fibrosclerosis, this variant is no longer classified as a variant of HT. Conversely, IgG4-related thyroiditis is now recognized as new entity of AITD (89). IgG4-related thyroiditis is associated with more frequent subclinical hypothyroidism and with higher levels of thyroid autoantibodies compared to the non-IgG4 thyroiditis group. In contrast to the other AITD forms there is a male preponderance (90). In long course of HT mainly IgG4 autoantibodies are produced (91), which trigger the development of IgG4-related disease (IgG4-RD). IgG4-RD can affect a variety of tissues (pancreas, skin, salivary glands, lacrimal glands, etc.), and the hallmarks are lymphoplasmacytic infiltrations with predominance of IgG4-positive plasma cells and fibrosis in the affected tissue. The atrophic form is more common than HT with enlargement of the thyroid gland (92). Although both forms lead to hypothyroidism, they have been reported as distinct diseases, differing in immunological background (associated with different HLA alleles), involvement of autoantibodies, and type of immune response (humoral versus cellular). The hypothesis that Ord’s disease was the end stage of HT could not be confirmed in follow-up studies (93). Thyroid autoantibody levels differ between goitrous and atrophic thyroiditis in that inhibitory TSHR antibodies are higher in Ord’s thyroiditis. These antibodies block cAMP production as well as TSH-induced DNA synthesis and iodide uptake (94). It has been hypothesized that antibody production promotes progression to hypothyroidism because higher levels of antibodies against Tg and TPO accompany deterioration of thyroid function (95). Destruction of the thyroid gland >90% leads to hypothyroidism (4). In the case of overt hypothyroidism, patients experience fatigue, weight gain, increased sensitivity to cold, difficulty concentrating, dry skin, nails, and hair, constipation, drowsiness, muscle soreness, and increased menstrual flow. HT is much more frequent in individuals affected by another AD. Although high anti-thyroid antibody titers may provide an indication of the likelihood of overt hypothyroidism, no correlation of antibody titer and risk for hypothyroidism has been found so far (96).

### Graves’ Disease

Symptoms often become manifest after emotional trauma and symptoms of hyperthyroidism arise with weight loss, weakness, dyspnea, palpitations, increased hunger and thirst, hyperdefecation, sweating, sensitivity to heat, tremor, irritability, and menstrual irregularity. Thyroid metabolism is accelerated with faster plasma turnover, higher TPO activity, excess Tg release, increased clearance of iodide from the plasma and decreased retention of iodide in the thyroid, and usually increased gland volume (97). Administration of high doses of I_2_ (1–2 mg) temporarily inhibits iodide uptake, trapping, organification, and hormone release in addition to reduction of thyroidal blood flow (98). Organification of I_2_ presumably is decreased by the blocking action of an oxidized iodide intermediate or depressed H_2_O_2_ generation. Although the blocking of I_2_ organification, reduced iodide uptake, and hormone release is a physiological reaction of the thyroid (Wolff–Chaikoff effect), it is more pronounced in GD patients than in healthy controls.

### Anti-thyroid Antibodies and Non-Thyroid Immunological Diseases

Circulating antibodies against thyroid antigens are not restricted to AITDs but were also detected in other common AD like RA, T1DM, and celiac disease (CD). Anti-TPO and anti-Tg antibodies were identified in 37 and 22.9% of RA patients, respectively. The authors detected anti-TPO in 13% and anti-Tg antibodies in 11.5% of healthy controls (99). In other studies lower prevalence of 16 and 12.3% in RA patients was detected, while the prevalence of anti-thyroid antibodies in healthy controls (8%) was similar (100, 101). Around 39.6% of T1DM patients had anti-TPO, and 30% had anti-Tg antibodies in one study, but other studies reported variations of 7–40% for anti-TPO and around 15% for anti-Tg antibodies (102, 103). This relatively high incidence in T1DM patients could not be explained by the fact that glycosylation of the Tg protein increased antigenicity or immunogenicity of the protein (104). Circulating anti-TPO and anti-Tg antibodies in CD patients have been detected in 11.7–30.5% (TPO) and 11.2–32% (Tg) (105) of patients. Other studies reported anti-thyroid antibodies in 26.2–41.1% of CD patients (106). The close interaction between CD and AITD was evidenced by the decrease of thyroid disorders and anti-thyroid antibodies after switching to a gluten-free diet. The link to other immune pathologies is further demonstrated by the fact that anti-TPO antibodies were also detected in patients affected by non-AD such as asthma patients. The antibodies were detected in 27.3% of subjects in one study and 15% of subjects in the other, compared to 10 and 8.3% in the controls (107, 108). Also 10–29% of idiopathic urticarial patients possessed anti-TPO antibodies, rather as an epiphenomenon than as cause of the disease (109). While the presence of anti-thyroid antibodies in other immune diseases is relatively frequent, patients with AITD produce antibodies against extra-thyroidal organs less frequently (10–15%) although comorbidities with other AD, such as vitiligo, may occur (110). It appears that subjects with deregulated immune systems are most prone to anti-thyroid antibody production. The prevalence of anti-TPO antibodies in AD is slightly higher than that of anti-Tg antibodies. In contrast to that, anti-TSHR antibodies have rarely been reported in non-thyroid immunological diseases. No expression of anti-TSHR antibodies was seen in RA patients while one study detected the antibodies in 18% of T1DM patients (111, 112). According to other authors, anti-TSHR antibodies are only seen in AITDs (21). The reason why anti-TSHR antibodies are specific for GD and antibodies against TPO and Tg also seen in other AD appears to suggest that HT and AD are more similar than GD and HT.

The link of specific pathological changes to anti-thyroid antibody titers in patients with other AD is complicated by the multifaceted manifestation of the diseases. Effects of anti-thyroid antibodies are best studied in subjects without AD or manifest thyroid dysfunction.

### Anti-thyroid Antibodies in Extra-Thyroidal Pathologies

**Figure 3:**
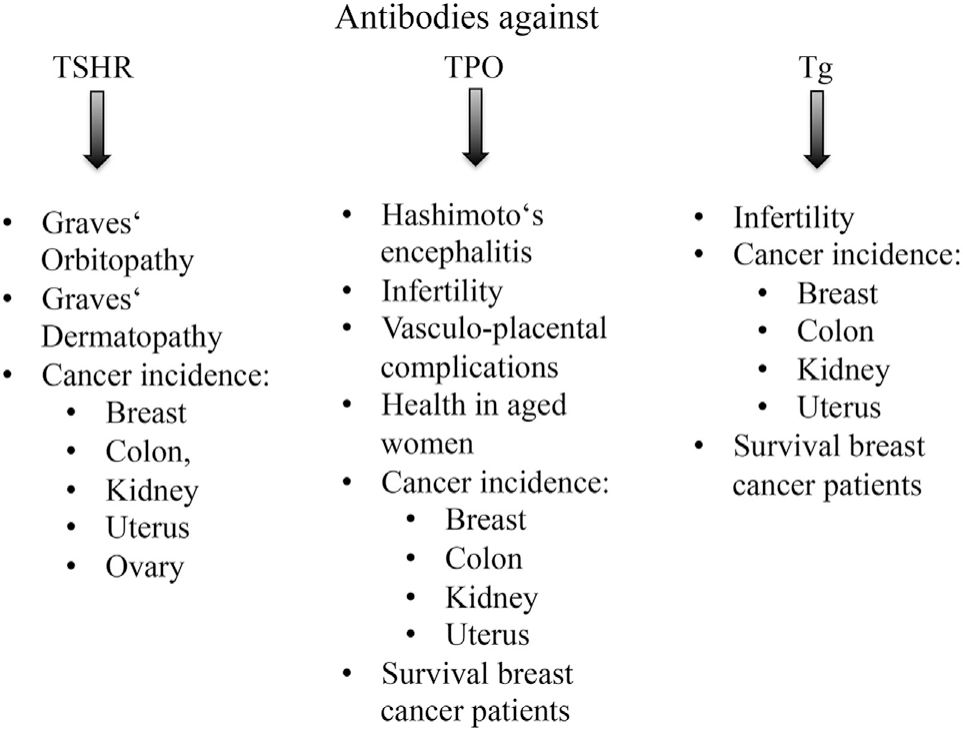
**Overview of extra-thyroidal effects of anti-thyroid antibodies**.

Extra-thyroidal effects of anti-TSHR, anti-Tg, and anti-TPO antibodies are shown in Figure 3.

#### Anti-TSHR Antibodies

**Figure 4:**
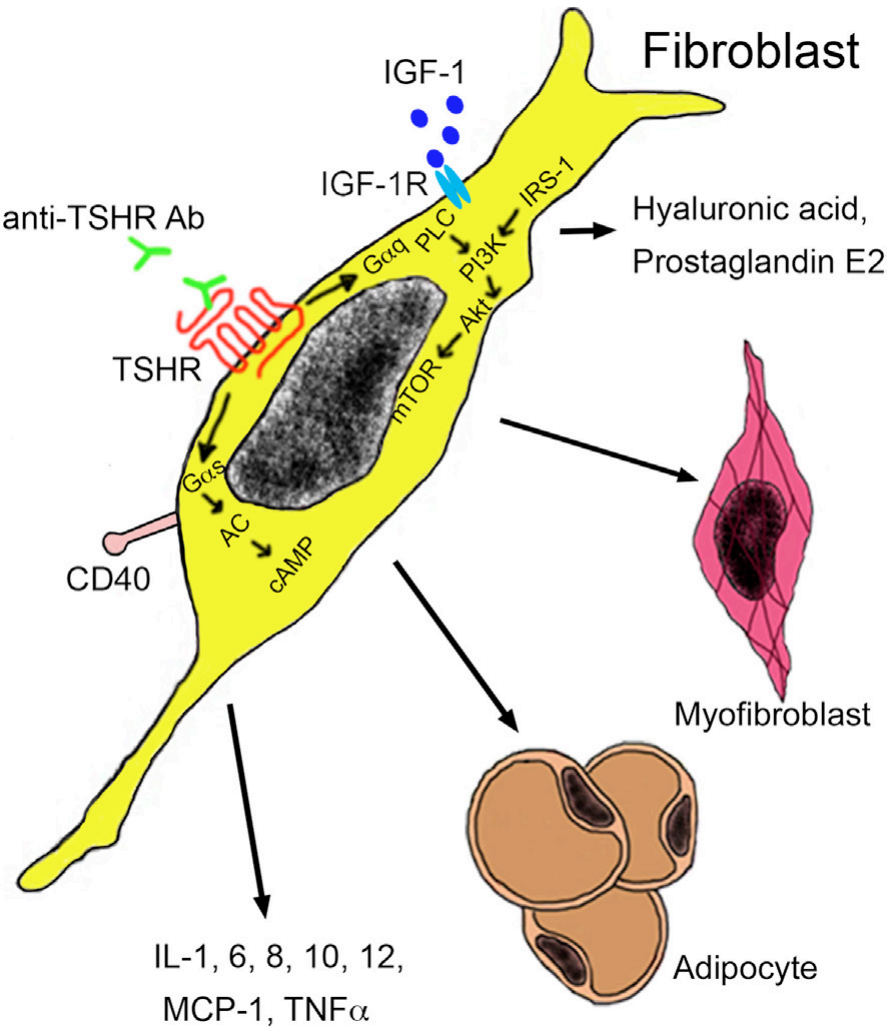
**Autoantibodies in the pathogenesis of Graves’ orbitopathy**. Anti-TSHR antibodies bind to the TSHR on orbital fibroblasts, which initiate signaling through Gαs and Gαq subunits. The Gαs complex initiates signaling by the protein kinase A through generation of cAMP by adenylyl kinase (AC). The Gαq pathway mediates activation of phospholipase C (PLC) and leads to activation of phosphatidylinositol 3,4,5 triphosphate by phosphatidylinositol 3,4,5 triphosphate kinase (PI3K) under the influence of insulin receptor substrate 1 (IRS-1). Akt activation results in mammalian target of rapamycin (mTOR) signaling with proliferation and secretion of cytokines, hyaluronic acid, and adipogenesis. Activation of orbital fibroblasts by T cells occurs *via* binding of the CD154 molecule on the T cell surface to the CD40 on the fibroblast surface. Abbreviations: IGF-1, insulin growth factor 1; IGF-1R, insulin growth factor 1 receptor; MCP-1, monocyte chemotactic protein 1; TNF-α, tumor necrosis factor-alpha; TSHR, thyroid-stimulating hormone receptor.

Extra-thyroidal manifestation of GD include swelling of the orbital tissue and of the skin, termed as *Graves’ orbitopathy* (*GO*) and *Graves’ dermopathy*. The term ophthalmopathy is also being used, but orbitopathy is preferred because it describes the pathological alterations more correctly. *GO* affects about 25% of GD patients (113) and results in dysmobility of extraocular muscles and optic nerve compression. It represents also the only extra-thyroidal pathology where the role of anti-thyroid antibodies is relatively well known. Target cells are infiltrating autoreactive T cells and fibroblasts resident between the extra-orbital muscles. These cell types interact *via* cytokine secretion and mutually stimulate each other. Both cell types express TSHR and IGF-1 receptors, which form a functional complex on orbital fibroblasts. Activated fibroblasts may either differentiate into myofibroblasts or adipocytes, or they may secrete hyaluronic acid and prostaglandin E2 (Figure 4). Major cytokines secreted by T cells include IL-1, IL-4, IL-6, TGF-β, leukoregulin, and IGF-1, while orbital fibroblasts release IL-1, IL-6, IL-8, IL-10, IL-12, MCP-1, and TNF-α (114). Inflammation and increased hyaluronic acid cause an increase in volume and lead to exopthalmos (115). Anti-TSHR antibody levels correlate with severity of GO and are predictive for the later development. By contrast, no correlation between severity of GO and hormone levels has been identified (116, 117). This finding highlights the role of TSHR antibodies as promoters of GO.

*Graves’ dermopathy* is an extra-thyroidal manifestation exclusively found in GD patients. Dermopathy develops in the presence of high TSHR antibody levels and is, with a frequency of 15%, a less common extra-thyroidal manifestation of GD (118). The main localization is pretibial and is associated with acropachy (digital clubbing, swelling of digits and toes, and periosteal reaction of extremity bones) in 20% of patients. TSHR is expressed in skin fibroblasts and abundant mucin deposition, possessing gel-like properties, separates the collagen fibers. Stimulation of fibroblasts by anti-TSHR antibodies along with mechanical factors and venous stasis causes accumulation of mucin. Trauma and injury may lead to the activation of T cells and the initiation of an antigen specific response, in this case the activation of fibroblasts and production of glucosaminoglycans (GAGs) (119).

#### Anti-TPO Antibodies

*Hashimoto’s encephalopathy* (*HE*) with confusion, agitation, seizures, status epilepticus, cognitive decline, and psychiatric symptoms appears to be independent of the state of the thyroid (120). HE is believed to be an immune-mediated disorder rather than representing the direct effect of an altered thyroid state on the central nervous system. Hypotheses include autoimmune vasculitis or other inflammatory processes, which may lead to disruption of the cerebral microvasculature. Although the presence of anti-TPO antibodies is the most common feature, a pathogenic role of anti-thyroid antibodies could not be identified (121). Further concerns about the relevance of anti-thyroid antibodies are linked to the use of assays that have not been validated for liquor as matrix. Matrix differences may play a role because liquor samples, due to the lower antibody levels that are expected, are usually tested in less diluted form than serum or plasma samples [for example, Ref. (122)]. Anti-TPO antibodies were shown to bind to cerebellar astrocytes, but their pathogenic role is unclear (123). Furthermore, α-enolase has been suggested as the true antigen for antibodies in HE (88). HT is also related to dysfunction of the vestibular system, which can be identified by abnormal caloric test and vestibular-evoked myogenic potential. The dysfunction was linked to increased anti-TPO antibodies without identification of the pathogenic mechanism (124).

Correlations of anti-thyroid antibody production with *decreased fertility* is suspected but was not confirmed in a recent study (125), which was performed in a highly selected study collective of predominantly very healthy and well educated women, and might not be representative for all women. The rate of miscarriage and preterm delivery was increased in women with anti-TPO and anti-Tg antibodies who underwent *in vitro* fertilization/intracytoplasmic sperm injection (IVF/ICSI). However, no difference was noted in the number of retrieved oocytes or success of IVF/ICSI (126). Anti-TPO antibodies are suspected to cause *vasculo-placental* complications, such as hypertonia, pre-eclampsia, preterm placenta abruption, postpartum hemorrhage, and postpartum thyroiditis (127). Since anti-thyroid antibodies can cross the placental barrier, they are able to induce hypothyroidism in the newborns. Women with high anti-TPO antibody titers display an impaired thyroidal response to human chorionic gonadotropin, and this may explain the higher risk of premature delivery in these women (128).

Positive *health effects* have been identified in older women. Women with anti-TPO antibodies were less frail, defined as weight loss >10% of body weight between 60 years and age at the evaluation, exhaustion, slowness, weakness, and low energy expenditure (129). The authors did not find any indications of deficient immune function in the non-seropositive (frailer) women. In contrast, they emphasized the concept of beneficial autoimmunity, which proposes that natural autoantibodies have a role in broadening the B-cell repertoire. Although this idea appears attractive, anti-thyroid antibodies do not fit into this classification because they do not bind any pro-inflammatory or cytotoxic mediators.

In summary, anti-TSHR antibodies affect eye and skin as part of GD, and the mechanisms of anti-TSHR antibody action is relatively well known, while effects of anti-TPO antibodies are more varied and affect central nervous system, female reproductive organs, and female health. The underlying mechanisms are largely unknown. Anti-Tg antibodies were often determined together with anti-TPO antibodies, but independent effects have been identified so far.

## AITD and Cancer

The risk of development of differentiated thyroid cancer (DTC) following AITD is still under debate. HT patients had a three times higher risk to develop papillary thyroid cancer according to some studies, while other studies did not report an increased incidence in HT patients (33). Differences in methodology, especially the use of fine needle biopsy versus thyroidectomy sections, might contribute to the contradictory results (130). While an increased risk has been identified based on evaluation of thyroidectomy samples [hazard ratio (HR) 1.15–4.16], fine needle analysis did not show such a link (HR 0.39–1.00). GD was associated with DTC with an adjusted HR of 10.4 for TC and explained by the stimulating activity of TSHR antibodies (131). Synchronicity of breast and thyroid cancer formation was found with the breast cancer growing faster (132).

Thyroid diseases, benign and malignant, presented an increased risk for extra-thyroidal cancers, particularly for breast, colon, melanoma, hematologic malignancies, uterus, kidney, and ovary (133). GD was associated with breast cancer with an HR of 1.54 (131). The main link between thyroid and breast cancer appears to occur *via* thyroid hormones. Breast cells possess dysregulated thyroid hormone receptors, and thyroid hormones can activate estrogen receptors, which correlate with breast cancer risk (133). High thyroid hormone levels were linked to breast cancer incidence, suggesting that the antibodies themselves were not important in the pro-cancer action. The study itself was conducted carefully, and the rate of mammography was not different between the groups. However, family history, smoking activity, and socioeconomic status were not evaluated. The coexistence of HT and TC has been reported at a rate of 1–23%, while correlation of lung and colon cancer and HT were reported only inconsistently. AITD was correlated to DTC risk, parathyroid tumors, oral, and breast cancer. Incidence of colon cancer, melanoma, and non-Hodgkin lymphoma was decreased (134, 135). Breast cancer prevalence was significantly higher in patients with thyroid diseases versus healthy controls (6.11 versus 2.07%). There was, however, no significant difference in the risk between type of thyroid disease or antibody status (136). AITD has been correlated with breast cancer prevalence in some cross-sectional studies (137–139). However, a correlation of thyroid diseases and breast cancer was documented in only 6 of 13 analyzed studies. Summarizing all studies, Sarlis et al. computed a relative risk for patients with thyroid diseases of 1.07 (140). The debate on the relationship of AITD and breast cancer is still not resolved, and indications of a higher percentage of thyroid dysfunction in breast cancer patients than in the general population are reported by some groups but not by others [for instance, Ref. (141–143)]. A major disadvantage of the cross-sectional studies is that breast cancer is already present, and an effect of breast cancer on thyroid physiology cannot be excluded. The best way to exclude such an influence is to analyze thyroid parameters and breast cancer incidence in prospective studies. Such studies have identified an association of high free thyroxine (fT4) and breast cancer incidence (144, 145). Elevated T3 levels were also correlated with a higher incidence of breast cancer as well as shorter survival time (HR 2.80) but not with increased overall cancer mortality (HR 1.09) (146, 147). In contrast, studies on free triiodothyronine (fT3) yielded discordant results on the link to breast cancer (145), and the authors advised that, due to the short half-life of fT3, either total T3 or fT4 were more suitable parameters for these analyses.

### Role of Anti-thyroid Antibodies

#### Cancer Incidence

The combination of high TSH levels, anti-TPO, and anti-Tg antibodies was identified as a risk factor for DTC (148), but seropositivity for Tg antibodies has not been reported consistently. An increased prevalence of melanoma, colon cancer, breast cancer, and hematological malignancies in patients suffering from GD or HT has been identified, but the overall risk for breast, colon, kidney, and uterine cancer was lower in patients with anti-TPO and anti-Tg antibodies than without (133). Prevalence of breast, colon, kidney, uterine, and ovarian cancer was increased in AITD patients with high titer anti-TSHR antibodies, but numbers were too low for statistical analysis. The authors identified a protective effect of anti-TPO and anti-Tg antibodies for breast cancer in this study and a previous study on the association of benign thyroid diseases and cancer (149). This correlation appears consistent with the finding that anti-TPO-positive breast cancer patients had better survival and longer disease-free intervals (150). Surprisingly, the beneficial action of anti-TPO antibody on breast cancer was restricted to women with thyroid volume (10–18 ml) in the normal range of between 10.7 and 17.5 ml (151–153). Several studies specifically investigated the role of anti-thyroid antibodies in breast cancer. In one study anti-TPO and anti-Tg seropositive patients had a higher risk of developing breast cancer than the antibody-negative group (154), while another study did not find such a correlation (136). Anti-TSHR antibody levels were higher in breast cancer patients than in women with benign tumors or healthy controls (155), but a pathogenic role of anti-TSHR antibodies in breast cancer is not expected because breast tissue does not express TSHR (156). According to time-dependency, antibody generation preceded the manifestation of breast cancer, making antibody production as an epiphenomenon of breast cancer unlikely (157). Seropositivity for TPO, but not for Tg, was linked to breast cancer prevalence, while no correlation was identified between the presence of anti-thyroid antibodies and tumor estrogen and progesterone receptor status (158, 159). A recent meta-analysis of eight studies reported higher anti-TPO and anti-Tg antibodies as well as higher T3 and T4 levels in breast cancer patients than in controls (160).

It might be speculated that inflammation initiated by anti-thyroid antibodies play an important role in promoting tumorigenesis. The fact that colon cancer is more frequent in patients with inflammatory bowel diseases than in controls supports the link of chronic inflammation and transformation (161). A causative role of chronic inflammation might also be deduced in breast cancer development since anti-TPO antibodies are able to cross-react with lactoperoxidase and initiate the inflammatory process. Cross-reactivity of anti-TPO antibodies with lactoperoxidase and myeloperoxidase as has been shown by Rapoport and McLachlan, and some anti-TPO antibodies actually inhibited peroxidase activity (162). Anti-TPO antibodies could also cause direct effects on neutrophilic granulocytes since its cross-reactivity with myeloperoxidase was observed in a subgroup of patients with vasculitic disorders and AITDs (163). Another study, however, failed to identify cross-reactivity of anti-TPO antibodies from AITD patients with myeloperoxidase (164).

In summary, a promoting role of anti-TSHR antibodies on breast cancer has been shown, while the role of anti-Tg and anti-TPO is unclear.

#### Breast Cancer Survival

The stronger link of thyroid disease to breast malignancies than to other cancers can be explained by the specific connection between these tissues. Twenty-eight percent of patients with benign fibrocytic mastopathy have anti-TPO antibodies, and 80% have thyroid hypertrophy. It is possible that generation of oxidative stress upon iodination of proteins may promote transformation of mammary cells and promote breast cancer development. On the other hand, I_2_ may have a beneficial role in tumor development; reduction of lipidperoxidation and formation of anti-proliferative lipids, iodolactones, has been reported (165). The intake of moderately high I_2_ concentrations (0.05% I_2_/kalium iodide) reduced mammary fibrosis in women by suppression of NIS, pendrin, TPO, and deiodinase type 1, but metabolism of I_2_ was found to be independent of TPO (166). Molecular I_2_ caused apoptosis in human breast cancer cells and reduced cancer growth in rat models (167). Consistent with this hypothesis, I_2_ levels in breast cancer were lower than in normal breast tissue (168).

Many studies recorded incidence without distinguishing between early and advanced breast cancer. In the study by Kuijpens et al., high TSH and anti-TPO antibody levels were not correlated with the risk of breast cancer (169). When patients were grouped into early and advanced stages, with the exception of an early study in 1976, thyroiditis was not linked to advanced breast cancer (138, 139, 170). When focusing on advanced stages of breast cancer, Fiore et al. identified a favorable effect of anti-TPO and/or anti-Tg antibodies on survival (171). Jiskra et al., on the other hand, found no influence of anti-thyroid antibodies on breast cancer survival (172). Thyroid hormones *per se* have an impact on breast cells by potentiating the growth-promoting effect of estrogen (173). Therefore, the presence of increased thyroid hormone levels impairs the identification of potential protective effects caused by anti-thyroid antibodies. A higher incidence of breast cancer in seropositive women could be explained by the fact that, due to the thyroid disease, these women received better medical care or had more frequent medical examinations, resulting in an earlier diagnosis of breast cancer. Since routine mammography has already been introduced in most countries, an earlier diagnosis due to stricter adherence to mammography would have to be suggested. There are, however, no indications that this could be the case (145). Studies on patients with newly diagnosed breast cancer reported that anti-TPO antibodies were linked to lower incidence of metastasis and less lymph node involvement (174–176). This finding might be explained by a better immune response against tumor tissue. On the other hand, longer survival of breast cancer patients with anti-thyroid antibodies could be due to the fact that these women are taking additional medications that might be beneficial for breast cancer.

There are currently no hypotheses as to how anti-thyroid antibodies can inhibit breast cancer progression. Despite correlations with lower proliferation rates and lower numbers of affected axillary lymph nodes, anti-TPO antibodies have not yet been proven to engage in interaction with receptors located on cancerous breast tissue (176). Therefore, it is assumed that they are simply indicators for a more active immune system, and that an active immune system may also limit progression of breast cancer. In contrast to that, anti-TSHR antibodies do not appear to have a positive effect in manifest breast cancer.

## Conclusion

There are currently only a few indications for a specific role of anti-thyroid antibodies in extra-thyroidal pathologies. Anti-TSHR antibodies differ from anti-TPO and anti-Tg antibodies in many respects (localization of the antigen, class, action, duration of increased levels, and prevalence of the antibody), which are indicated in Table 1. Most importantly, action of anti-TSHR antibodies appears relatively well defined and restricted to specific targets (skin, eye), while action of the other anti-thyroid antibodies is less well known and less specific. The role of thyroid diseases and anti-TPO and anti-Tg antibodies in the development of breast cancer has been investigated in numerous studies with still controversial results. Reasons for this include the use of cross-sectional studies where breast cancer was diagnosed and a potential influence of breast cancer cannot be ruled out. Anti-TPO antibodies might initiate chronic inflammation and destruction of mammary cells *via* cross-reactivity with lactoperoxidase, but generation of oxidative stress by lactoperoxidase activity alone might also promote breast cancer. Due to the known interaction of thyroid hormones and estrogen on breast cells, abnormal hormone levels in manifest AITD may aggravate or mask the effect of anti-thyroid antibodies. Anti-thyroid antibodies may also display an effect on cancer progression, but early and advanced breast cancers in several studies were not analyzed separately. More precise data on the promoting or protecting action of anti-thyroid antibodies have been obtained by prospective studies and by distinguishing between early and advanced breast cancer. These studies appear to indicate that anti-TPO antibodies might have a beneficial effect in manifest breast cancer. In addition to other factors, these results are influenced by different time points of blood sampling, either before or after breast surgery, and recruitment of patients in third level oncologic centers (143). However, different effects of anti-thyroid antibodies in both cancer development and progression could explain the contradictory findings. Such behavior has already been shown for autophagy in breast cancer (177). Autophagy is reduced in the development of breast cancer but increased in tumor progression when nutrients and oxygen supply become limiting factors for growth. Anti-thyroid antibodies in breast cancer may not have a beneficial action *per se* but may be indicators for a more active immune system. The finding that older women with anti-thyroid antibodies are less fragile than the seronegative control group may support this theory.

Although GD and HT belong both to AITD, they have different pathogenic mechanisms. The absence of cytotoxicity in GD is a major difference to HT and other AD. Location and immunogenicity of the antigen (178) and dominant class of the antibodies may contribute to these differences. Surprisingly, most of the contributing factors (Figure 1) are similar for both AITDs.

## Author Contributions

EF and RW planned the review outline and wrote the manuscript.

## Conflict of Interest Statement

The authors declare that the research was conducted in the absence of any commercial or financial relationships that could be construed as a potential conflict of interest.
